# Registered nurses’ perspective of nurse practitioners: A mixed‐methods study

**DOI:** 10.1111/inr.13102

**Published:** 2025-02-19

**Authors:** Tatyana Miller, Joshua Porat‐Dahlerbruch, Shoshana Ratz, Moriah E. Ellen

**Affiliations:** ^1^ Department of Health Policy and Management Faculty of Health Sciences & Guilford Glazer Faculty of Business Ben‐Gurion University of the Negev Beer‐Sheva Israel; ^2^ Herzog Medical Center Jerusalem Israel; ^3^ Department of Acute & Tertiary Care School of Nursing University of Pittsburgh Pittsburgh Pennsylvania USA; ^4^ Israel Implementation Science and Policy Engagement Centre Ben‐Gurion University of the Negev Beer‐Sheva Israel

**Keywords:** Attitude of health personnel, health policy, healthcare workforce, interprofessional relationships, nurse practitioners, organizational policy, registered nurses

## Abstract

**Aim:**

To understand what registered nurses (RNs) know about the nurse practitioner (NP) role, and factors affecting RN perspectives toward NPs.

**Background/Introduction:**

One issue hindering the integration of NPs into healthcare systems is poor RN–NP relationships. This relationship has been understudied where the NP role has been recently introduced.

**Methods:**

This study used an explanatory sequential design. A cross‐sectional survey was disseminated to RNs to identify knowledge and feelings regarding NPs and factors influencing their perspectives. Based on survey results, semistructured interviews were conducted and analyzed using thematic analysis. Quantitative and qualitative results were integrated to identify converging, diverging, and complementary results. This study adheres to the Checklist of Mixed‐Methods Elements in a Submission to Advance the Methodology of Mixed‐Methods Research.

**Results:**

There were 277 survey respondents. The factors impacting perspectives toward NPs were age, exposure to NPs, years of experience, and level of education. Seven themes arose from the qualitative data: exposure to NPs, delineation of NP scope of practice, characterizations of NPs, acceptance of the role, advantages of NPs, cultural aspects, and effects of higher education. Quantitative and qualitative results converged in nearly all instances.

**Discussion:**

To improve the RN–NP relationship, RNs must understand the NP role. RNs who have worked directly with NPs usually express the benefits of NP care for patients and care team members.

**Conclusion:**

To promote the integration of NPs into care teams, it is important to expose RNs to the NP role through education or work experience.

**Implications for nursing policy:**

Upstream approaches to improve the RN–NP relationship include educating students about the NP role, offering clinical rotations with NPs, and organizational messaging promoting the RN–NP collaboration.

## INTRODUCTION

A nurse practitioner (NP) is defined by the International Council of Nurses as “an advanced practice nurse who integrates clinical skills associated with nursing and medicine to assess, diagnose and manage patients in primary healthcare settings and acute care populations, as well as ongoing care for populations with chronic illness” (Schober, 2020). The NP workforce is growing rapidly worldwide. In the United States, the Bureau of Labor Statistics (2024) predicts that the NP workforce will grow 45% by the year 2032, making it one of the fastest‐growing occupations. Rapid NP workforce growth in many countries significantly outpaces growth in the physician workforce (Maier & Aiken, 2016).

In Israel, the Ministry of Health aims for 6% of all RNs to be NPs by 2030 (Fighel & Hefetz, 2023). This goal poses policy challenges for two primary reasons: (1) There are insufficient positions available to employ all newly licensed NPs; and (2) there are concerns that attracting nurses to NP roles will worsen the existing nursing shortage (Porat‐Dahlerbruch et al., 2023b). However, to provide improved care and outcomes to patients, it is not sufficient to increase the number of NPs; NPs must be fully and properly integrated into the healthcare system to achieve the best possible outcomes (Porat‐Dahlerbruch et al., 2023a). To achieve optimal integration, all relevant stakeholders involved in integrating NPs must be considered (Andersen et al., 2021). Research has been conducted on physicians' viewpoints and patient attitudes toward NPs. However, there has been scant research on RNs’ perceptions of NPs.

## BACKGROUND

NPs occupy a unique position, trained in nursing but given authorities historically reserved for physicians. These authorities include evaluating patients, ordering and interpreting diagnostic tests, and prescribing medications (Depriest et al., 2020). This valuable combination of skills and authorities, stemming from NPs’ patient‐, community‐, and family‐centered approach, facilitates a holistic approach to care and benefits organizations employing NPs, interprofessional care teams, patients, and the health system overall (Aiken et al., 2021).

Positive patient outcomes linked to NP care have been reported (McMenamin et al., 2023). There is growing evidence that NPs can reduce costs and physician workload while maintaining high quality of care and patient satisfaction (Suzuki et al., 2022). The addition of NPs to the care team was linked to improved communication and care coordination for patients with complex needs (Lowe et al., 2018).

In response to these potential positive outcomes associated with NP care internationally, many health systems have strived to increase the size of the workforce. For instance, in 2022, 8% of all registered nurses in the United States, and 2.8% of all registered nurses in Canada were NPs. In Israel, fewer than 1% of RNs are NPs. Percentages are low in other countries as well (e.g., England (1.2%), Scotland (1.6%), Netherlands (2.3%), Australia (0.7%), New Zealand (1%), and Japan (0.04%) (Brownwood & Lafortune, 2024).

Despite the low percentage of RNs licensed as NPs in Israel, the Ministry of Health aims to increase the percentage to 6% by 2030 (Fighel & Hefetz, 2023). There are many advantages to integrating more NPs into the health system, such as professionalization of nursing, filling provider shortages, and achieving improved patient outcomes. However, rapid integration of NPs without proper infrastructure in place can lead to worsening RN shortages and lower quality of didactic and clinical education (Porat‐Dahlerbruch et al., 2023b). Furthermore, resistance to the NP role often ensues; political and health system battles can result in a limited scope of NP practice in the field (Torrens et al., 2020). Relationships between NPs and other health professionals, such as RNs, are critical to ensuring that countries not only reach their goals of growing the NP workforce, but that NPs are also functioning as intended. Otherwise, ambitious policies, such as Israel's 6% by 2030 goal, may lead to resource waste.

### Integration challenges

NP integration is defined as “a multilevel process of incorporating NPs into the health care system to an extent at which they can function to the full extent of their education, training, and scope of practice and contribute to patient, health system, and population needs” (Porat‐Dahlerbruch et al., 2022). Expert opinion defines NP integration as a complex process requiring constant revision of policy at national, jurisdictional, and organizational levels (Porat‐Dahlerbruch et al., 2023a). Ineffective NP integration policies have been tied to a waste of resources and discourages RNs from pursuing the NP role (Porat‐Dahlerbruch et al., 2022).

One of the most challenging issues entailed in integrating NPs is the rapid pace at which the health system hierarchy changes (Porat‐Dahlerbruch et al., 2023a). Nurses are given expanded authority overlapping with the traditional role of physicians. Maier and Aiken (2016) termed this phenomenon as “task shifting.” There is little international agreement on the exact appropriate tasks for NPs (Rioux‐Dubois & Perron, 2022). This can result in poor working relationships, macro‐level political tension between professional stakeholder groups, professional posturing, and a rise in overall resentment (Dickman et al., 2018). Unlike in many countries where managers establish positions in the health system, in Israel, most open positions are established by government allotment, known as a “teken.” As such, macro‐level policy in Israel plays a particularly large role.

Poor NP–RN relationships also affect NP integration. A 2015 study from Australia described overt and covert hostilities from RNs toward NPs, including passive–aggressive behaviors, dismissive behaviors, and outright obstruction. In return, NPs have characterized their RN colleagues as “the enemy within” (Maclellan et al., 2015). A study in Norway found that RNs perceived NPs as a threat to existing organizational structures. RNs expressed concern that NPs would take all the “interesting” tasks, leaving them with more mundane work. However, many RNs were unclear as to the actual scope of the NP role (Boman et al., 2018). This illustrates the importance of introducing NPs into care teams gradually, allowing for care team members to become accustomed to the idea of working with NPs, prepared for their entrance into the workforce, and perhaps even formed a positive view of future collaborations (Contandriopoulos et al., 2015). A recent study in Israel investigated stakeholders perceptions toward NPs, including physicians, NPs themselves, administrators, and policy makers, though did not include the viewpoint of the RNs (Porat‐Dahlerbruch et al., 2023); this study aims to fill that gap in knowledge.

Setting fixed NP percentage goals, such as Israel's 6% goal, does little to mitigate the risks of rapid NP integration. As more and more countries aim to expand their NP workforce (Brownwood & Lafortune, 2024) in an attempt to improve provider availability and patient outcomes, they run the risk of detrimental outcomes such as physician resistance, RN pushback, and exacerbation of RN shortages (Dickman et al., 2018).

Evidence suggests that barriers to integration, such as poor NP–RN relationships, can be overcome through a systematic plan of integration at both national and organizational levels (Porat‐Dahlerbruch et al., 2023a). To create an effective integration plan, it is vital to form a clear understanding of the factors affecting the NP–RN relationship. This is particularly important in countries recently introducing the NP role, such as Israel (Porat‐Dahlerbruch et al., 2023a). Yet, as of now, there is no standardized, structured framework for the process of integrating NPs.

### Aim of the study

This study aims to fill the gaps in knowledge regarding NP integration policy by improving our understanding of the factors affecting RN perceptions of NPs.

## METHODS

### Study design

This mixed‐methods study used an explanatory sequential design. In explanatory sequential designs, a qualitative strand follows a quantitative strand in efforts to explain findings. Here, a quantitative survey was followed by one‐to‐one interviews. We selected this design for several reasons. First, it was expected that further data would be needed to clarify relationships identified in the survey. Second, part of the goal was to identify groups on which to focus during interviews (i.e., particular RN characteristics driving significant and nonsignificant results). Third, the research problem is more quantitatively oriented, thus rendering an initial survey appropriate for facilitating creating an interview guide. Fourth, the researchers had the time and ability to return to participants for richer, qualitative data (Creswell & Plano Clark, 2017). The first stage aimed to identify and document factors affecting RN perceptions toward NPs. To gain deeper insights into these factors, one‐to‐one interviews with a subset of participants were conducted. We integrated data at two points—between the quantitative and qualitative strands to produce initial results, as well as after the qualitative strand to explain initial results from the quantitative strand (Supporting Information Material S1) (Creswell & Plano Clark, 2017).

### Phase 1: Quantitative strand

#### Sample and setting

This study was conducted in Israel and involved RNs throughout the country. We recruited participants for both phases through Israeli nursing Facebook groups. The use of Facebook groups allowed for rapid and extensive dissemination of surveys at a low cost, allowing us to reach more participants (Schneider & Harknett, 2022). To be included as a participant in either study phase, participants were required to self‐identify as an RN currently working in Israel and to be fluent in either Hebrew or English. NPs and retirees were excluded. A power analysis calculation was conducted to understand how many respondents were needed for the survey. For a power of 80%, we needed a sample size of at least 99, and for a power of 90%, we needed a sample size of at least 132.

#### Data collection

Despite an extensive review of the literature, using multiple scholarly search engines, no validated survey on the relationship between NPs and RNs was found. We instead developed a survey based on evidence gathered from the literature and personal experience. To enhance construct validity, an advisory panel of NPs, RNs, and experts in the field reviewed the survey (Bowen‐Mendoza et al., 2022). The final survey consisted of nine demographic questions and 18 questions (Supporting Information Material S2).

#### Statistical analysis

We used descriptive statistics to analyze the survey data according to demographic categories—i.e., sex, age, religious affiliation, years of experience, education level, work setting, location of employment, and whether they have worked with NPs. We performed chi‐squared tests to determine if there is a correlation between demographic categories and the respondent's attitudes toward NPs.

The survey respondents were dichotomized: those who worked with NPs and those who did not. Using *t* tests, we compared the responses of these two groups to determine whether there is a difference in the level of knowledge about NP education requirements and scope of practice.

We used bivariate linear regression to determine if there was a correlation between RN demographics and responses to the NP knowledge questions. For regressions, RN demographics were divided into bivariate categories, as is often done to enhance interpretability (Aiken et al., 2021).

#### Validity and reliability

To demonstrate the reliability and validity of survey questions, content, cognitive, and usability standards were applied (Groves et al., 2009). Content standards were met through expert review of survey questions. A committee of three nursing workforce experts in Israel and two methodology experts ensured that the questions measured the intended concepts. Cognitive and usability standards were met by piloting and accordingly revising the survey. We further tested the validity of the survey by comparing responses from groups expected to report differing results. For example, RNs who had worked and not worked with NPs had statistically different responses on their understanding of the NP scope of practice (*p* = 0.0003). Reliability of the survey was assessed using Cronbach's alpha, which was “acceptable” or “good” at 0.75 (Groves et al., 2009). Further, response bias is a concern in survey research, especially with social media surveys (Schneider & Harknett, 2022). To mitigate this risk, we compared the demographics of the respondents to national RN demographics and found a high degree of similarity (Haron et al., 2014).

### Phase 2: Qualitative strand

#### Sample and recruitment

Convenience and snowball sampling were used (Leighton et al., 2021) to recruit RNs who represented a subset of participants. Participants were recruited by word of mouth. We aimed to reach RNs who have and have not worked with NPs to achieve a broad range of perspectives. Interviews were conducted until the point of data saturation (Mwita, 2022).

#### Data collection

We used the results from Phase 1 to construct a semistructured interview guide. The interview guide consisted of demographic questions, 17 interview questions, and probing questions (Supporting Information Material S3). The interviews were conducted by an NP researcher and recorded using the Zoom application at a time and place of the interviewee's convenience. Interviews lasted an average of 30 minutes. Interviews were conducted over a period of one month.

#### Qualitative analysis

The recordings were transcribed. The transcripts were reviewed for accuracy, and any identifying information was removed. Inductive content analysis was used (Proudfoot, 2023). Two coders first read the interviews to familiarize themselves with the data. They then identified keywords and concepts within the data and created a preliminary codebook. They compared the preliminary codes and agreed on a final codebook. After coding the full transcripts independently, they compared the codes. A third research team member resolved any discrepancies. Together, the research team identified recurring concepts and induced themes.

#### Rigor

Measures were taken to ensure rigor. Coding was conducted independently. The NP interviewer conducted peer debriefing with non‐NP study team members to elucidate and prevent any bias (Janesick, 2007, pp. 1–2). Agreement on themes was reached by consensus, and data from both phases of the study were integrated to obtain more reliable and dependable results (Thomas & Magilvy, 2011).

#### Integration of quantitative and qualitative

Integration is used to enhance rigor by identifying converging, diverging, and complementary results between quantitative and qualitative strands (Campbell et al., 2020). We used the qualitative results to enhance our understanding of the quantitative data.

### Ethical considerations

Ethical approval was given by Ben‐Gurion University's Human Subjects Research Committee for both the quantitative and qualitative strands (ME28092022 and ME01032023, respectively).

## RESULTS

### Phase 1: Quantitative strand

#### Survey respondents

There were 277 respondents who completed the survey (Table 1). About half of the respondents previously worked with NPs. Ninety‐two percent of RNs were female. About 90% of respondents were Jewish and 37% worked in the peripheries. These demographics were similar to the national RN population (Haron et al., 2014).

**TABLE 1:** Survey respondent demographics.

Demographic	Category	Count	Percentage
Works with NP	Yes	142	51%
	No	135	49%
Gender	Female	256	92%
	Male	21	8%
Religion	Jewish	249	90%
	Not Jewish	28	10%
Age, years	≤44	170	61%
	≥45	107	39%
Place of education	In Israel	259	94%
	Outside of Israel	18	6%
Highest education level	BSN with or without post‐BSN certificate	156	56%
	MSN or higher	121	44%
Work experience, years	≤10	118	43%
	≥11	159	57%
Location of work	Urban	175	63%
	Periphery	102	37%
Workplace	Outpatient	155	56%
	Inpatient	122	44%

#### Descriptive statistics

Mean responses to each item are in Supporting Information Material S4. RNs were amenable to NPs and felt that they were qualified practitioners. Many would consider training to become NPs in the future. Most RNs reported comfort carrying out orders from NPs.

#### Multiple regressions

We conducted bivariate regressions using mean responses for each statement (dependent variables) and RN characteristics (independent variables) (Table 2). We ran ordered logistic regressions with the dependent variable (i.e., whether the RN had worked with an NP), while controlling for the highest level of education, age, and years of experience.

**TABLE 2:** Odds ratios estimating responses to statements based on work experience with NPs.

	Model 1	Model 2
	Estimated separately and unadjusted, OR (95% CI)	Estimated separately and adjusted, OR (95% CI)
I know the licensing process for becoming an NP in Israel.	1.628 [1.071–2.476]^*^	1.641 [1.073–2.509]^*^
I understand the full scope of practice of NPs in Israel.	2.168 [1.418–3.313]^***^	2.165 [1.409–3.33]^***^
I feel that the addition of NPs to the medical system will confuse patients.	0.635 [0.417–0.966]^*^	0.646 [0.422–0.988]^*^
I feel that NPs should be allowed to order lab tests.	1.905 [1.183–3.066]^**^	2.157 [1.317–3.534]^**^
I would personally prefer to be treated by a doctor as opposed to an NP.	0.629 [0.413–0.957]^*^	0.602 [0.393–0.922]^*^
I would be welcoming NPs in my workplace.	1.79 [1.128–2.839]^*^	2 [1.244–3.217]^**^
I would feel uncomfortable taking orders from an NP.	0.419 [0.27–0.65]^***^	0.398 [0.255–0.621]^***^

^*^ *p* < 0.05;

^**^ *p* < 0.01;

^***^ *p* < 0.001.

*Note*: Model 2 is adjusted for the highest level of education, age, and years of work experience of the registered nurse.

RNs who had worked with NPs rated their knowledge of the NP licensing process (OR = 1.641; CI: 1.073–2.509; *p* = 0.023) and NP scope of practice higher (OR = 2.165; CI: 1.409–3.33; *p* = 0.000). They were less likely to: (1) feel that adding NPs to the health system would confuse patients (OR = 0.646; CI: 0.422–0.988; *p* = 0.034), (2) prefer treatment from physicians than NPs (OR = 0.602; CI: 0.393–0.922; *p* = 0.020), and (3) feel uncomfortable taking orders from an NP (OR = 0.398; OR: 0.255–0.621; *p* = 0.000).

We ran regressions for the mean responses based on the highest level of education (Table 3). First, we ran bivariate regressions with each independent variable. Then, we ran ordered logistic regressions, accounting for previous work experience with NPs, age, and years of experience.

**TABLE 3:** Odds ratios estimating responses to statements based on highest level of education.

	Model 1	Model 2
	Estimated separately and unadjusted, OR (95% CI)	Estimated separately and adjusted, OR (95% CI)
I know the requirements for becoming an NP in Israel.	1.57 [1.23–2.005]^***^	1.45 [1.098 –1.915]^**^
I know the licensing process for becoming an NP in Israel.	1.951 [1.525–2.497]^***^	1.914 [1.441–2.544]^***^
I understand the full scope of practice of NPs in Israel.	1.707 [1.343–2.17]^***^	1.587 [1.205–2.092}^***^
I feel that NPs can help resolve the physician shortage.	1.279 [1.007–1.625]^*^	1.366 [1.046–1.783]^*^
I feel that NPs should be allowed to prescribe medications.	1.319 [1.032–1.685]^*^	1.571 [1.187–2.08]^*^
I feel that NPs should be allowed to order lab tests.	1.325 [1.018–1.725]^*^	1.604 [1.172–2.194]^**^
I feel that the development of the NP role allowed for career advancement for nurses.	1.179 [0.909–1.527]	1.354 [1.009–1.816]^*^

^*^ *p* < 0.05;

^**^ *p* < 0.01;

^***^ *p* < 0.001.

*Note*: Model 2 is adjusted for the highest level of education, age, and years of work experience of the registered nurse.

RNs who held a master's degree or higher rated their understanding of the requirements to become an NP (OR = 1.45; CI: 1.098–1.915; *p* = 0.000), NP licensing process (OR = 1.914; CI: 1.441–2.544; *p* = 0.000), and NP scope of practice (OR = 1.587; CI: 1.205–2.092; *p* = 0.000) higher. They felt more strongly that NPs could help resolve the physician shortage (OR = 1.366; CI: 1.046–1.783; *p* = 0.043) and that NPs should be authorized to prescribe medications (OR = 1.571; CI: 1.187–2.08 *p* = 0.027) and order lab tests (OR = 1.604; CI: 1.172–2.194; *p* = 0.037). They felt that the addition of the NP to the profession facilitates career advancement for nurses (OR = 1.354; CI: 1.009–1.816; *p* = 0.049).

### Phase 2: Qualitative interviews

#### Participant characteristics

We interviewed 10 RNs aged 30 to 52 years. Ninety percent were female, and 10% were male. Years of RN work experience ranged from 2 to 35 years. Among participants, 60% and 40% worked in inpatient and outpatient settings, respectively. Eighty percent of the participants were Jewish. Forty percent of the participants had worked with NPs. Regarding the highest level of education, one participant had an associate degree, five had bachelor's degrees, three had post‐baccalaureate certificates, and one had a master's degree.

#### Themes

Seven themes arose from the interviews: exposure to NPs, NP role definition and delineation of the scope of practice, characterizations of NPs, acceptance of NPs, advantages of incorporating NPs into the health system, Israeli culture, and effects of higher education of RNs (Supporting Information Material S5).

#### Exposure to NPs

RNs who had worked with NPs had a greater understanding of the benefits of incorporating NPs into the health system. RNs who had not worked with NPs were more hesitant to acknowledge the benefits. All participants felt that the experience of working with NPs would help elucidate the importance of incorporating NPs into the health system and their value to the care team. RNs who had worked with NPs stated that prior to working with NPs, they did not understand the potential value of incorporating NPs into the healthcare system. This was the most commonly recurring theme.

*Truthfully before I started working here, I didn't know nurse practitioners. I did not know what all of that was. So, after I became familiar and started working with them, of course, it really makes a difference, if we work with them*.

#### NP role definition and delineation of the scope of practice

There was some confusion among RNs regarding the definition and scope of the NP role. Participants felt that there was no clear and consistent job description for NPs in most organizations. They did not understand NP authorities. They felt that NPs themselves might lack clarity of their own scope of practice. Many NPs were perceived as having created their own role as opposed to following a defined one. RNs questioned whether NPs are intended to function as physicians or traditional nurses.

RNs also expressed concerns that patients might be confused by the concept of NPs. Participants noted that patients already have difficulty differentiating staff members, physicians, RNs, and custodial staff—adding an additional role could exacerbate the confusion.

*They'll do what a lot of the doctors do, but then at some point it's like, wait a minute, you're also a nurse and where do you fall? So, it's a little confusing as to what they are*.

#### Characterizations of NPs

Participants characterized NPs as highly educated. Since the education program is arduous, participants expressed respect for the NPs’ achievements. The participants noted that medical knowledge alone is not sufficient to provide the ideal patient‐centered care needs. Participants who had worked with NPs stated that the integration of a holistic, patient‐centered and traditional medical approach to care engrained in NPs can be advantageous compared to physicians. Participants also perceived NPs as having superior communication skills.

*If you can have the education of a doctor with the empathy that really comes with being a nurse, I think that a patient could get a much higher level of care*.

#### Acceptance of NPs

Participants felt that NPs expanded the skills associated with the nursing profession. They felt that NPs were one of them or part of the nursing profession. NPs themselves worked as RNs in the past—when NPs give an order, they understand how it will affect the RN. While all participants noted that they personally welcome working with NPs, they thought that some RNs may feel threatened by the NP role.

*You still get the same medical care but with the nurse's perspective and eye, which in a way is kind of the perfection that we're working [towards] with patient centered care*.

#### Advantages of incorporating NPs into the health system

RNs recognized certain advantages to working with NPs. Due to their higher level of education, NPs have greater knowledge and a broader scope of practice. They feel that NPs are attentive and readily available to answer questions from both the care team and patients. While physicians were perceived as attempting to avoid the RNs’ questions, NPs were happy to address them. RNs appreciated that NPs reasoned their conclusions, fostering learning. Participants felt that NPs could help address the physician shortage.

*When you put together the knowledge of the doctor in them with the nurse in them together, it makes me trust them more than I do some of our doctors*.

#### Israeli healthcare work culture

RNs described the Israeli health system work culture as hierarchical. There are levels of RN status that are typically based on years of work experience, which can lead to confusion as to where NPs fall into the hierarchy. Although NPs may have fewer years of work experience than some RNs, they are given more responsibility and respect. This is a disruption of the status quo. RNs thought that there was an inverse relationship between age and the ability to accept changes in this hierarchy. Therefore, they felt that older nurses might be less accepting of NPs than younger nurses.

*We're not really sure where exactly they fall into the hierarchy. It is a little confusing. There's nothing that says ‘this is exactly what a nurse practitioner is’*.

#### Effects of higher education on the RN's perception of the NP

Participants felt that there was a positive relationship between RN education level and exposure to NPs. Additionally, they believed that higher levels of education would facilitate a better understanding of the NP role since RNs who are more academically inclined would be more likely to pursue the NP role.

*The higher up you are, the more registered you are, the more you have your advanced degree or whatever else, the more knowledge you have, and the more you talk about it*.

#### Integration of quantitative and qualitative results

The qualitative and quantitative data converged in many instances. The data showed that RN exposure correlates positively with the degree of openness to the NP role. However, the analysis also showed that RNs were concerned that adding NPs into the health system might confuse patients. The data converged to show that RNs were not confident that they understood the NP scope of practice. In both strands, the data converged to show that the NP role allows advancement and expansion of the nursing role and that RNs would welcome the incorporation of NPs into the health system. RNs felt that NPs could fill in the gaps created by the shortage of physicians in the health system. Survey results showed that older RNs would be less welcoming of NPs compared to their younger counterparts, and the interviews reinforced this finding. The qualitative and quantitative data converged to show that RNs who had a higher level of education understood the NP role better.

The qualitative and quantitative data sometimes complemented each other. The quantitative data showed that NPs have advantages over physicians when it comes to treating patients, and the qualitative data showed that NPs combine their medical knowledge with their nursing skills to provide patient‐centered care. The qualitative data also demonstrated that NPs augment the workforce by bringing personable traits, generally associated with nurses, to traditional medical roles.

The qualitative and quantitative data diverged regarding the correlation between higher RN levels of education and degree of exposure to NPs. The interviewed RNs felt that RNs with a higher level of education would have a greater level of exposure to NPs, though the survey results showed that level of education made little difference in RN degree of exposure to NPs.

## DISCUSSION

The purpose of this mixed‐methods study was (a) to examine how familiar RNs in Israel are with the NP licensing process and scope of practice, (b) to describe RN perceptions toward NPs, and (c) to determine whether there is a correlation between various characteristics of RNs and their perceptions of NPs. We found that RNs somewhat understood the NP licensing process and scope of practice. However, the overall NP role definition and scope of practice were less clear. RNs generally had a positive attitude toward the integration of the NP role into the health system. The two factors that most affected RNs’ perceptions of NPs were whether they had worked with NPs in the past and their highest level of education.

### RNs’ knowledge of the NP role

This study revealed that RNs felt minimally familiar with the licensing process for NPs in Israel. RNs with a higher level of education were more confident in their knowledge. This is consistent with other studies correlating exposure with knowledge (Porat‐Dahlerbruch et al., 2023b). There is a general lack of understanding of the NP role worldwide (Rioux‐Dubois & Perron, 2022). International research has found that many do not understand whether NPs function more as physicians or nurses (Maier & Aiken, 2016). This confusion arose frequently in our interviews as well.

A 2023 expert opinion paper on NP integration discussed the necessary steps to implementing policy changes regarding NP integration at multiple health system levels (Porat‐Dahlerbruch et al., 2023a). At the macro level, the national government needs to create policies clearly defining the NP scope of practice. Once in place, at the meso level, organizational policy must facilitate NP practice to the full extent of their scope, as well as their education and capabilities. On the micro level, the care team members must fully understand and implement the interface between members of the care team and the NP. This includes physicians, RNs, and, of course, the NPs themselves. Still, this “trickle‐down process” of NP integration would not work efficaciously without a clear, macrothislevel role definition and a general understanding of the role.

This study showed that RNs with a higher level of education rated their understanding of the NP scope of practice higher than those with a lower level of education. This is consistent with a similar study conducted in Israel in 2018 showing that nurses who held academic degrees expressed more positive attitudes toward expanding nursing scope of practice (Dickman et al., 2018). If RNs do not fully grasp the NP role, they will have less amenability to working with NPs or becoming NPs themselves, rendering Israel's 6% NP goal unattainable and unsustainable. Adding information about the NP role to standard RN curricula in the formative years may pave the way for the better acceptance of the NP role.

### Acceptance of NPs

In our survey, we asked RNs how welcoming they would be of NPs in their place of work. The opinions were extreme. Out of 277 respondents, not one gave a moderate response—all responses were strongly disagree, disagree, agree, or strongly agree. Demographics that correlated with these results were age, level of education, and previous work experience with NPs. The influence of RN characteristics has been found to affect their perceptions of NPs throughout the international literature (Jedwab et al., 2022; Ylitörmänen et al., 2023). While most RNs reported openness to NPs, it is essential to understand the skepticism of those who were opposed. Understanding why particular RNs feel the way that they do will help design interventions targeted to assuage skepticism. This concept can be applied to other countries designing policies to integrate and expand the NP workforce (Porat‐Dahlerbruch et al., 2023a).

### Perceptions of NPs

Our study found that RNs perceived NPs as providing high‐quality, holistic care to patients. Specifically, NPs combine their nursing skills with medical knowledge and are more accessible and attentive to them. These views were consistent with previous research conducted both in Israel (Dickman et al., 2018) and internationally (Ruiz, 2020).

Most RNs reported feeling comfortable taking orders from NPs. They understood that the NPs’ higher level of education enables the scope of practice expansion. Additionally, NPs have worked as nurses in the past, which was mentioned as a reason for more familiarity with and respectfulness toward RNs. The phenomenon of feeling that the NP is “one of them” has been documented in multiple countries (Lane, 2020).

### NPs fill gaps in the healthcare system

RNs felt that NPs could alleviate the physician shortage. RNs felt that NPs provide quality medical care equal to those of physicians in many cases. Physician shortages are a worldwide concern that is worsening as the population ages (Cooper et al., 2017). This healthcare provider shortage may be even worse in developing countries (Al Ismaili et al., 2024). NPs can be leveraged to improve care delivery. For example, NPs can help alleviate the workload pressure on physicians, reduce wait times, and provide patient‐centered care (Brownwood & Lafortune, 2024). NPs can be utilized internationally to help fill this gap (Torrens et al., 2020). Adding to the NP workforce, while creating proper infrastructure for NP integration, can provide the ideal solution to provider shortages.

### NPs are strong communicators

This study showed that NPs were perceived as “team players” and good communicators. They were more readily accessible than physicians and were considered to be more open to answering questions presented by either RNs or patients. This has been documented in prior studies with patients (Vinall‐Collier et al., 2016) and RNs (Rayo et al., 2014). NPs have been found to help bridge the communication barrier between physicians and RNs, resulting in better multidisciplinary collaboration and better patient outcomes (Matziou et al., 2014).

## LIMITATIONS

One limitation of the study is the use of a non‐validated survey. The questions used for this survey were not empirically tested but were developed based on theory and work experience within the Israeli health system. We attempted to mitigate this limitation and enhance content validity by receiving input from an expert advisory panel (Bowen‐Mendoza et al., 2022). Moreover, the converging themes found in the methodological integration of quantitative and qualitative findings can be considered a form of member checking to help validate the findings (Campbell et al., 2020). Both phases of this study used a convenience sample, obtained from Facebook groups which might have increased the risk of sample bias. To mitigate this issue, we aimed to reach RNs from varying demographic groups to more accurately reflect the current population of RNs (Haron et al., 2014; Schneider & Harknett, 2022).

## CONCLUSION AND RECOMMENDATIONS

Promoting positive working relationships between RNs and NPs can help integrate the new NP role. Age, years of work experience, education level, and previous work experience with NPs impact the relationship between NPs and RNs. Resources would be best invested in policy affecting the most modifiable factor—i.e., RN work experience with NPs. Work experience promotes understanding of the NP role and its benefits to the health system. In turn, RNs are likely to be more accepting of the NP role in practice and advance in the career path to become an NP. The latter is particularly critical for meeting goals to increase the size of the NP workforce, such as Israel's 6% by 2030 goal. Two policy options to promote RN work experience with NPs and promote integration are employing NPs in various settings and instating education about the NP role in curricula in the formative years. Nevertheless, increasing the size of the NP workforce is beneficial only if there is a sufficient supply of NP positions to employ new graduates and NPs are integrated sufficiently enough to practice to the full extent of their education and scope of practice.

## IMPLICATIONS

Efficacious integration of NPs into the health system requires the acceptance and/or cooperation of RNs. Exposure to NPs has been shown to be the primary modifiable factor addressable through policy interventions. Both government and organizational policymakers should aim to insert NPs in as many settings as possible, both within the hospital framework and in primary care to facilitate greater exposure of RNs to NPs. Educating RNs about the NP role in the formative years of education would also help promote the NP role. Either of these policy interventions would help countries reach their goal of growing the NP workforce to fill provider shortages and improve care quality.

### Upstream policy recommendations

Upstream strategies for promulgating the NP role are particularly important in countries with centralized health system planning. One upstream approach is mandating curricula to include RN student exposure to the NP role. Practical examples would include discussing the NP role as part of the foundations of the profession course and offering a clinical rotation shadowing an NP. Our results and other studies show that the more exposure that RNs get to NPs during the formative years, the more RNs will accept and be attracted to the NP role (Porat‐Dahlerbruch et al., 2023b, 2024).

Another non‐education program option would be a national marketing campaign to expose healthcare workers and the public to the NP role. Government bodies can allocate tuition for NP programs and incentivize employing care organizations by offsetting costs for missed workdays due to schooling. Furthermore, creating collaboration opportunities between professional nursing bodies can create a feeling of unity and support among the diverse nursing roles. These policy recommendations are supported by recent studies conducted in Israel (Porat‐Dahlerbruch et al., 2023b, 2024) and internationally (Torrens et al., 2020).

## AUTHOR CONTRIBUTIONS

*Conceptualization*: Tatyana Miller and Joshua Porat‐Dahlerbruch. *Methodology*: Tatyana Miller, Joshua Porat‐Dahlerbruch, and Moriah E. Ellen. *Software*: Tatyana Miller and Joshua Porat‐Dahlerbruch. *Formal analysis*: Tatyana Miller, Joshua Porat‐Dahlerbruch, and Shoshana Ratz. *Investigation*: Tatyana Miller. *Data curation*: Tatyana Miller. *Writing—original draft*: Tatyana Miller. *Writing—review and editing*: Joshua Porat‐Dahlerbruch, Moriah E. Ellen, and Shoshana Ratz. *Visualization*: Tatyana Miller. *Resources*: Joshua Porat‐Dahlerbruch and Moriah E. Ellen. *Project administration*: Tatyana Miller and Joshua Porat‐Dahlerbruch. *Supervision*: Joshua Porat‐Dahlerbruch and Moriah E. Ellen. *Validation*: Joshua Porat‐Dahlerbruch and Moriah E. Ellen.

## CONFLICT OF INTEREST STATEMENT

No conflict of interest has been declared by the authors.

## FUNDING INFORMATION

This research received no specific grant from any funding agency in the public, commercial, or not‐for‐profit sector.

## Supporting information



Supporting information

Supporting information

Supporting information

Supporting information

Supporting information
