# Supplementary material for: Registered nurses’ perspective of nurse practitioners: A mixed‐methods study
Source: Int Nurs Rev. 2025 Feb 19;72(1):e13102. doi: 10.1111/inr.13102 (PMC11921077; doi:10.1111/inr.13102)
Supplement: Supplementary file 1 — Supporting information [file INR-72-0-s005.docx]

**Supplementary Material 1: Mixed-Methods Study Design**

QUANT

Qual

Interpretation of results
