# Supplementary material for: Registered nurses’ perspective of nurse practitioners: A mixed‐methods study
Source: Int Nurs Rev. 2025 Feb 19;72(1):e13102. doi: 10.1111/inr.13102 (PMC11921077; doi:10.1111/inr.13102)
Supplement: Supplementary file 2 — Supporting information [file INR-72-0-s002.docx]

Supplementary Material 2: Questionnaire

Hello,

My name is [removed for peer review]. I am a Geriatric Nurse Practitioner, and I am currently conducting research as part of a thesis at [removed for peer review].

We are conducting this study in order to understand what registered nurses in Israel know and feel about nurse practitioners in the Israeli healthcare system. The knowledge that will be acquired from this research will hopefully aid in the integration process of nurse practitioners in the health system in Israel. This study is open to all registered nurses currently working in Israel.

Your participation in this study is voluntary and anonymous. You do not have to participate in this study, and you can leave at any time. The benefit of your participation in this study is assistance in the integration process of clinical specialist nurses in the health system in Israel. There is no risk in participating in the study. The study was approved by the research ethics committee at Ben-Gurion University.

The process includes filling out an online questionnaire, which takes 3-5 minutes. The answer will be anonymous and no identifying information such as your name, email address, or IP address will be collected. All information collected will be stored on [removed for peer review]'s secure servers.

If you have any questions about the research, you can contact me at [removed for peer review].

Before continuing, please answer the following question: Are you a registered nurse in Israel?

1. Yes
2. No

Please answer the following questions:

1. Sex
   1. Male
   2. Female
   3. Other
2. Age
   1. Up to 29
   2. 30-44
   3. 45-54
   4. 55- 66
   5. 67+
3. Religious affiliation
   1. Jewish
   2. Muslim
   3. Christian
   4. Other
4. Years of work experience
   1. Under 5
   2. 6-10
   3. 11-15
   4. 16-20
   5. 20+
5. Education level in Nursing, check all that apply
   1. Practical nurse
   2. BSN
   3. Post-BSN certification
   4. MSN
   5. PHD or another doctorate
6. Where did you earn your nursing degree?
   1. Israel
   2. Out of Israel
7. Have you ever directly worked with a nurse practitioner?
   1. Yes
   2. No
8. What is your main place of employment?
   1. Community care
   2. Medical center
   3. Hospital
   4. Other
9. In what area do you currently work?
   1. Jerusalem
   2. Center
   3. Haifa / North
   4. Judea and Samaria
   5. South

Please mark how you feel about the following statements on a scale from “strongly agree” to “strongly disagree”.

|  | Strongly  agree (1) | Agree (2) | Somewhat Agree  (3) | Niether agree  not disagree (4) | Somewhat disagree  (5) | Disagree (6) | Strongly  Disagree (7) |
| --- | --- | --- | --- | --- | --- | --- | --- |
| I know the requirements for becoming an NP in Israel. |  |  |  |  |  |  |  |
| I know the full licensing process for becoming an NP in Israel. |  |  |  |  |  |  |  |
| I know the full scope of practice of NPs in Israel. |  |  |  |  |  |  |  |
| I feel that having NPs in the medical system is a positive development. |  |  |  |  |  |  |  |
| I feel that NPs can help resolve the physician shortage. |  |  |  |  |  |  |  |
| I feel that NPs can help bridge the workflow gap between physicians and nurses. |  |  |  |  |  |  |  |
| I feel that the addition of NPs to the medical system will confuse patients. |  |  |  |  |  |  |  |
| I feel that NPS are qualified enough to diagnose patients. |  |  |  |  |  |  |  |
| I feel that NPs are qualified enough to treat patients. |  |  |  |  |  |  |  |
| I feel that NPs should be allowed to prescribe medications. |  |  |  |  |  |  |  |
| I feel that NPs should be allowed to order lab tests. |  |  |  |  |  |  |  |
| I would personally prefer to be treated by a physician as opposed to an NP. |  |  |  |  |  |  |  |
| I would be welcoming of NPs in my workplace. |  |  |  |  |  |  |  |
| I feel that NPs have advantages over physicians when it comes to treating patients. |  |  |  |  |  |  |  |
| I feel that the development of the NP role allows for career advancement nurses. |  |  |  |  |  |  |  |
| I feel that my knowledge is underutilized in the workplace. |  |  |  |  |  |  |  |
| I would feel uncomfortable taking orders from a NP. |  |  |  |  |  |  |  |
| I would consider becoming an NP in the future |  |  |  |  |  |  |  |

Thank you for your participation in this study; this will help us further the integrations of NPs into the Israeli health system.
