# Supplementary material for: Registered nurses’ perspective of nurse practitioners: A mixed‐methods study
Source: Int Nurs Rev. 2025 Feb 19;72(1):e13102. doi: 10.1111/inr.13102 (PMC11921077; doi:10.1111/inr.13102)
Supplement: Supplementary file 3 — Supporting information [file INR-72-0-s004.docx]

Supplementary Material 3: Interview Guide

Opening statement:

Today, I will be facilitating an interview to understand your perspectives regarding registered nurse's perceptions of Nurse Practitioners in Israel. We will synthesize the themes from these interviews and eventually report results.

Any self-identifying information from this interview will be removed from all transcripts and reported results. You may cease to participate at any time. Do you have any questions? (Ask them to sign the informed consent) I will ask a list of questions from a pre-prepared interview guide along with follow-up, clarifying questions. Please let me know if any questions are unclear.

Demographic Questions:

1. Where do you work now?
2. Where have you worked in the past?
3. How many years have you had as a registered nurse?
4. How old are you?
5. What religion do you identify as?
6. What is your highest level of education *in nursing*? Interview questions:
   1. Have you worked with an NP in the past? If yes, how were your interactions?
   2. How do you feel about NPs entering the Israeli healthcare system?
   3. What are your thoughts about the quality of care provided by NPs?
   4. How do you think that your nursing colleagues feel toward NPs? What drives these thoughts?
   5. We found that registered nurses who have worked with NPs were more likely to rate their knowledge of the licensing process required to become an NP higher than those who have not, why do you think that is?
   6. We found that registered nurses who had a higher level of education were more likely to rate their knowledge of the licensing process required to become an NP higher than those who have not, why do you think that is?
   7. We found that registered nurses who have worked with NPs were more likely to rate their knowledge of the scope of practice of NP higher than those who have not, why do you think that is?
   8. We found that registered nurses who had a higher level of education were more likely to rate their knowledge of the licensing process required to become an NP higher than those who have not, why do you think that is?
   9. We found that registered nurses who have not worked with NPs think that adding NPs for the medical system will confuse patients more than those who have worked with NPs, why do you think that is?
   10. We found that registered nurses who have worked with NPs would be less likely to prefer being treated by a doctor as opposed an NP, why do you think that is?
   11. We found that registered nurses who have worked with NPs, would be more welcoming of NPs in their workplace, as opposed to those who have not, why do you think that is?
   12. We found that registered nurses who have worked with NPs, felt more comfortable taking orders from NPs as opposed to those who have not, why do you think that is?
   13. Nurses who had a higher level of education also rated their understanding of the licensing process and scope of practice of NP higher as opposed to those with a lower level of education. Why do you think that is?
   14. Nurses who had a higher level rated that they felt that NPs should be allowed to prescribe medications and order lab tests higher, as opposed to those with a lower level of education. Why do you think that is?
   15. Nurses who had a higher level rated that they felt that the NP role allows for greater opportunities for career advancement of nurses higher, as opposed to those with a lower level of education. Why do you think that is?
   16. Do you think that the age of the registered nurse would affect how they feel about NPs?
   17. Do you think that the years of work experience of the registered nurse would affect how they feel about NPs?

Closing statement:

Thank you for your time. Your expertise on this subject matter will go a long way in helping understand the registered nurse and nurse practitioner relationship. Is it ok if I follow up over the phone if we have any additional questions?
