# Supplementary material for: Registered nurses’ perspective of nurse practitioners: A mixed‐methods study
Source: Int Nurs Rev. 2025 Feb 19;72(1):e13102. doi: 10.1111/inr.13102 (PMC11921077; doi:10.1111/inr.13102)
Supplement: Supplementary file 4 — Supporting information [file INR-72-0-s003.docx]

Supplementary Material 4: Mean Responses and Standard Deviations for Survey Responses

| **Statement** | **Mean Response** | **Standard Deviation** |
| --- | --- | --- |
| I know the requirements for becoming an NP in Israel. | 5.32 | 1.8 |
| I know the licensing process for becoming an NP in Israel. | 4.84 | 1.9 |
| I understand the full scope of practice of NPs in Israel. | 4.29 | 1.9 |
| I feel that having NPs in the medical system is a positive development. | 6.08 | 1.8 |
| I feel that NPs can help resolve the doctor shortage. | 5.35 | 1.8 |
| I feel the NPs can help bridge the workflow gap between physicians and nurses. | 5.13 | 1.8 |
| I feel that the addition of NPs to the medical system will confuse patients. | 3.56 | 1.3 |
| I feel that NPs are qualified enough to diagnose patients. | 5.68 | 1.3 |
| I feel that NPs are qualified enough to treat patients. | 6.06 | 1.1 |
| I feel that NPs should be allowed to prescribe medications. | 5.69 | 1.5 |
| I feel that NPs should be allowed to order lab tests. | 6.34 | 1.1 |
| I would personally prefer to be treated by a physician as opposed to an NP. | 3.47 | 1.8 |
| I would be welcoming of NPs in my workplace. | 6.26 | 1.1 |
| I feel that NPs have advantages over physicians when it comes to treating patients. | 5.51 | 1.7 |
| I feel that the development of the NP role allows for career advancement for nurses. | 6.22 | 1.3 |
| I feel that my knowledge is underutilized in the workplace. | 4.29 | 2.0 |
| I would feel uncomfortable taking orders from an NP. | 2.34 | 1.4 |
| I would consider becoming an NP in the future. | 5.09 | 2.0 |

**CAPTION**: N=277. Responses are on a Likert scale of 1-7( 1= strongly disagree to 7 = strongly agree). N=277
