# Supplementary material for: Registered nurses’ perspective of nurse practitioners: A mixed‐methods study
Source: Int Nurs Rev. 2025 Feb 19;72(1):e13102. doi: 10.1111/inr.13102 (PMC11921077; doi:10.1111/inr.13102)
Supplement: Supplementary file 5 — Supporting information [file INR-72-0-s001.docx]

**Supplementary Material 5**: Synthesis of Results—Converging, Diverging, and Complementary Themes from Qualitative and Quantitative Strands

| Qualitative Theme | Description of Qualitative Theme | Qualitative Direct Evidence | Quantitative Result | Quantitative Direct Evidence | Conclusion |
| --- | --- | --- | --- | --- | --- |
| Exposure to NPs | RNs who were exposed to NPs had positive feelings towards NPs entering the Israeli Healthcare system as opposed to RNs who have not be exposed to NPs who were more hesitant of the idea. | “We work with them. We see how wonderful they are and how helpful it is to have them.”  “I feel like most people just wouldn’t know what to expect because we haven’t been exposed to it. No one’s really been exposed to nurse practitioners.” | RNs who had work experience with NPs were more welcoming of NP’s entering their workplace as opposed to those who have not. | I would be welcoming of NPs in my workplace. ^1^  OR [95% CI] 2.0  [1.25-3.22] p-  value <0.01 | The qualitative and quantitative data converge to show that exposure of RNs to NPs correlates with being more open to the NP role being added to the healthcare system. |
|  | Exposing RNs to NPs can help make them understand the importance of adding NPs to the healthcare system | “Truthfully before I started working here I didn’t know nurse practitioners. I did not know what all of that was. So after I became familiar and started working with them, of course, it really makes a difference, if we work with  them.” |  |  |  |

| Qualitative Theme | Description of Qualitative Theme | Qualitative Direct Evidence | Quantitative Result | Quantitative Direct Evidence | Conclusion |
| --- | --- | --- | --- | --- | --- |
| Delineating NP Role: Definition & Scope of Practice | There is no clearly defined job description of the NP role in most organizations. | “They’ll do what a lot of the doctors do, but then at some point it’s like, wait a minute, you’re also a nurse and where do you fall? So it’s a little confusing as to what they are.” | RNs felt that the addition of NPs into the health system may confused patients although others felt that it would not be an issue. | I feel that the addition of NPs into the health system will confuse patients. Rated 3.56 out of a 7-point Likert scale with a SD of 1.8. ^1^ | The qualitative and quantitative data converge to show that RNs fear that the addition of NPs into the healthcare system may confuse patients. |
|  | There is confusion regarding the role of NPs, there is a lack of clarity as to whether they work as physicians or traditional nurses. | “We are not sure if they are more like the doctors, they are more like the nurses, they are somewhere  between.” | RNs were not confident in their knowledge of the full scope of practice of NPs. | I know the full scope of practice of NPs in Israel.  4.29 out of a 7- point Likert scale with a SD of 1.9.^1^ | The qualitative and quantitative data converge to show that RNs were not confident in their knowledge of NPs the full scope of practice. |
|  | There is a concern  about patient confusion regarding the NP role. | “The patients  will probably be confused. They sometimes call the nurse “doctor” that is already sign that they do not exactly understand how the healthcare  system works.” |  |  |  |
|  | NPs themselves  are confused regarding their own scope of practice | “I think they  themselves are… slightly confused as to what their job is supposed to be.” |  |  |  |

| Qualitative Theme | Description of Qualitative Theme | Qualitative Direct Evidence | Quantitative Result | Quantitative Direct Evidence | Conclusion |
| --- | --- | --- | --- | --- | --- |
| Characterizations of NPs | NPs are associated with providing holistic, patient- centered care. | “I think the quality of care provided is a very high quality because it is a mix, especially in geriatrics. It is a mix of the nursing and the medicine it brings in the “peopleness”, the humane part of the care, as well as  the medical.” | RNs felt that NPs are qualified enough to diagnose patients. | I feel that NPs are qualified enough to diagnose patients.  Rated 5.68 out of a 7-point Likert scale with a SD of 1.3. ^1^ | The qualitative and quantitative data complement each other. The quantitative data showed that NPs have advantages over physicians when it comes to treating patients and the qualitative data showed that NPs combine their medical knowledge with their nursing skills to provide patient centered care. |
|  | NPs are highly educated | “It was a really long course. It was a very difficult process.” |  |  |  |
|  | NPs are knowledgeable about medicine. | “I think their knowledge is more than ours is.” |  |  |  |
|  | NPs are highly educated | “They’ve been working really hard to get where they are.” |  |  |  |
|  | Some aspects of the NP approach to care are advantageous when compared to physicians because they combine medical knowledge with the holistic patient care model. | “If you can have the education of a doctor with the empathy that really comes with being a nurse, I think that a patient could get much higher level of care.” | RNs felt that NPs had advantages over physicians when it comes to the treatment of patients. | I feel that NPs have advantages over physicians when it comes to treating patients. |  |
|  |  |  | RNs did not prefer to be treated by a physician as opposed to an NP. | I would personally prefer to be treated by a doctor as opposed to an NP. Rated 3.47 out of a 7-point Likert scale with a SD of 1.8. ^1^ |  |

| Qualitative Theme | Description of Qualitative  Theme | Qualitative Direct Evidence | Quantitative Result | Quantitative Direct Evidence | Conclusion |
| --- | --- | --- | --- | --- | --- |
| Acceptance of NPs | The interviewed RNs believe that NPs have expanded the scope and skills of the nursing profession. | “You still get the same medical care but with the nurse’s perspective and eye which, in a way, kind of the perfection that we’re working with patient  centered care.” | RNs felt that the NP role allows for career advancement for NPs. | I feel that the development of the NP role allows for career advancement for nurses.  Rated 6.21 out of a 7-point Likert scale with a SD of 1.3. 1 | The qualitative and quantitative data converge to show that the NP role allows for expansion and advancement for RNs and that RNs would be welcoming to the addition of NPs into the healthcare systems. |
|  | The interviewed RNs felt that NPs are one of them. | “NPs were once one of us.” |  |  |  |
|  | The interviewed RNs felt welcoming of NPs but thought that those who do not understand what NPs are may feel threatened by them. | “From what I see and experience. I find that we all like them. We all respect them.”  “Threatened because they don’t necessarily know what their position becomes.  Threatened because up until now, up until nurse practitioners in a way, the number of years they were a nurse was your  standing.” | RNs felt that they would be very welcoming of NP’s in the healthcare system. | I would be welcoming of NPs in my workplace.  Rated 6.26 out of a 7-point Likert scale with a SD of 1.1. ^1^ |  |

| Qualitative Theme | Description of Qualitative  Theme | Qualitative Direct Evidence | Quantitative Result | Quantitative Direct Evidence | Conclusion |
| --- | --- | --- | --- | --- | --- |
| Advantages of NPs | NPs have an expanded knowledge level due to their higher level of education. | “They have done a lot more learning and studying, I think their knowledge is more than ours  is. |  |  | The qualitative and quantitative data converge to show that RNs felt that NPs could fill in the gap in the healthcare system created by a shortage of physicians. The qualitative data also complements the quantitative data by showing that NP’s augment the workforce by bringing nursing personable traits to |
|  | NPs have a wider scope of practice due to their higher education. | “A person who studies, who goes deep into certain disciplines, and they pass tests which are administered by regulated authorities, which can grant authorizations to write scripts, they know what they are doing. You see their competence level, and you see what they’re capable of doing and you understand what their education is.” |  |  |  |
|  | NPs help fill the gap in the healthcare system created by the physician shortage. | “We all know that the health system here is socialized medical care, which means there is a lot of pressure on healthcare and there are not enough doctors…and to have the NP around we’ll make it much easier.” | RNs felt that NPs could help fill the healthcare gap created by the physician shortage. | I feel that NPs can help resolve the physician shortage.  Rated 5.35 out of a 7-point Likert scale with a SD of 1.8. ^1^ |  |
|  |  |  | RNs thought that NPs could help fill the workflow gap between physicians and nurses. | I feel that nurses NPs can help bridge the workflow gap between doctors and nurses.  Rated 5.13 out of a 7-point Likert  scale with a SD of 1.8. ^1^ |  |

|  | NPs are readily available to answer questions from the staff as well as the patients. | “We would not have to deal with accessing the doctor that does not want to be  accessed.” |  |  | the traditional medical roles. |
| --- | --- | --- | --- | --- | --- |
|  | NPs are attentive. | “By and large  the NPs generally speak more to their patients and listen  more.” |  |  |  |
|  | NPs provide  holistic care that can help provide better patient outcomes. | “You’re more  likely as a nurse practitioner, to see the human as human, as a person, not just the medical.” |  |  |  |
|  | RNs feel that they can trust NPs | “When you put together the knowledge of the doctor in them with the nurse in them together, it makes me trust them more than I do some of our doctors.” |  |  |  |

| Qualitative Theme | Description of Qualitative  Theme | Qualitative Direct Evidence | Quantitative Result | Quantitative Direct Evidence | Conclusion |
| --- | --- | --- | --- | --- | --- |
| Israeli Healthcare Work Culture | The Israeli healthcare system is described as hierarchal. | “That again will go back to hierarchy, the  doctor is everything.” | There was a statistically significant difference in response to the question “I would be welcoming of NPs in my workplace.” based on age. | RNs up to age 44 had an average response of 6.38 and those over age 45 have an average response of 6.06, this difference statistically significant with a chi2 p- value of 0.03. | The qualitative and quantitative data converge to show that the older RNs would be less welcoming of NPs as opposed to their younger counterparts. |
|  | There is confusing  regarding where NPs fall into the traditional health system hierarchy. | “We’re not  really sure where exactly, they fall into the hierarchy. It is a little confusing.  There’s nothing that says this is exactly what a nurse  practitioner is.” |  |  |  |
|  | There is an inverse  relationship between age and acceptability of change in the hierarchical health system structure. | “Those with  more seniority are older and have a more difficult time accepting new things.”  “The younger generation is more hungry for information, for more to do, for less bedside nursing and more knowledge and  more medical nursing.” |  |  |  |

| Qualitative Theme | Description of Qualitative Theme | Qualitative Direct Evidence | Quantitative Result | Quantitative Direct Evidence | Conclusion |
| --- | --- | --- | --- | --- | --- |
| Effects of Higher Education of the Registered Nurse | RNs who had a higher level of education had more exposure to NPs. | “The higher up you are, the more registered you are, the more you have your advanced degree or whatever else, the more knowledge you have, and the more you talk about it. | There was no statistical significance between RNs who had a higher or lower level of education regarding whether they have worked with an NP. | Compared responses of RNs who have a lower education level  Chi2 p-value 0.95 | The qualitative and quantitative data diverge in terms of the correlation between higher levels of education of the RN and exposure to NPs. The interviewed RNs felt that those with a higher level of education would have a greater level of exposure to NPs. However, survey result show that while a higher percentage of RNs who have a higher level of education have worked with NPs this difference  was not statistically significant. |
|  | RNs who had a higher level of education understood the NP role better. | “Because they are working toward a higher level of practice already. Again, they are academic minded, they are more career focused. They have seen the higher level of practice, at least to some degree.” | RNs who had a higher level of education rated their knowledge of the requirement for becoming an NP, the licensing process of becoming an NP and the full scope of practice of NPs higher as opposed to RNs with a lower level of education. | RNs who had a higher level of education rated their knowledge of the requirements for becoming an NP in Israel higher as opposed to nurses with a level of education.  OR [95% CI]  1.45 [1.098-  1.915] p-value  <0.01  RNs who had a higher level of education rated their knowledge of the licensing process for  becoming an NP in Israel higher | The qualitative and quantitative data converge to show that RNs who had a higher level of education under the NP role better than those with a lower level of education. |

|  |  |  |  | as opposed to nurses with a level of education.  OR [95% CI]  1.914 [1.441-  2.544] p-value  <0.001  RNs who had a higher level of education rated their knowledge of the full scope of practice of NPs in Israel higher as opposed to nurses with a level of education.  OR [95% CI]  1.587 [1.205-  2.092] p-value  <0.01 |  |
| --- | --- | --- | --- | --- | --- |

**CAPTION:** All quantitative results are based on a 7-point Likert scale (1=Strongly Disagree; 7= Strongly Agree). Each qualitative theme is linked to converging, diverging, and complementary results from the quantitative strand. Conclusions according to each qualitative theme are listed.
